# Safety Assessment of Foods and Drinks Consumed by People on a Gluten-Free Diet

**DOI:** 10.3390/molecules27196165

**Published:** 2022-09-20

**Authors:** Anna Przybylska, Agnieszka Chrustek, Beata Sperkowska, Marcin Koba, Dorota Olszewska-Słonina

**Affiliations:** 1 Department of Toxicology and Bromatology, Faculty of Pharmacy, L. Rydygier Collegium Medicum in Bydgoszcz, Nicolaus Copernicus University in Torun, A. Jurasza 2 Street, 85089 Bydgoszcz, Poland; 2 Department of Pathobiochemistry and Clinical Chemistry, Faculty of Pharmacy, L. Rydygier Collegium Medicum in Bydgoszcz, Nicolaus Copernicus University in Torun, M. Curie-Skłodowskiej 9 Street, 85094 Bydgoszcz, Poland

**Keywords:** gluten-free food, naturally gluten-free food, ELISA, beer

## Abstract

Naturally gluten-free foods and processed foods that do not contain information about the potential presence of gluten in them pose a hypothetical threat to people with food allergies and celiac disease. Patients who should follow a strict gluten-free diet do not always do so. Therefore, the aim of this research was to analyze certified “gluten-free” and naturally gluten-free products without labeled “may contain gluten” information in terms of their content of gluten proteins. The enzyme immunoassay AgraQuant Gluten G12 ELISA test kit was used for the analysis. Of all the products used in the research, only 5.8% were found to contain gluten above 20 ppm. Only one product labeled “gluten-free” was contaminated with gluten at 79.3 ppm (cider cake). In addition, our research also examined the gluten content of commercial beers containing barley malt not labeled as “gluten-free”. Research has shown that 60% of samples are not safe for those on a strict gluten-free diet. Our research clearly shows that many manufacturers, although they do not monitor their products for the presence of gluten in them, offer safe products, although they cannot be recommended in a gluten-free diet. Therefore, there is a strong need to increase the frequency of testing by food manufacturers for the presence of gluten in their products, so that the number of products approved for people on a gluten-free diet continues to increase.

## 1. Introduction

The Food and Agriculture Organization of the United Nations (FAO) states that the basis of food security is access to safe and valuable food. Food quality and safety must be controlled at all stages of its production, from the producer to the consumer. This guarantees the repeatability of the products in terms of health requirements. A gluten-free diet (GFD) is a special diet that eliminates foods containing wheat, rye and barley proteins, which was first introduced in 1941 by Willem Karl Dicke [1]. This diet recommends eating unprocessed foods that are naturally gluten-free, such as fruits, vegetables, meat, eggs and fish [2,3], but also foods that are certified as “gluten-free”. Gluten is the protein fraction found in wheat (gliadin), rye (secalin), barley (hordein) or their cross varieties and their derivatives [4,5]. The immunogenic fractions of gluten proteins are gliadins and glutenins [6]. It should be mentioned that, according to the Codex Alimentarius, a product can be labelled “gluten-free” when it contains less than 20 ppm of gluten [7] and “ultra-low-gluten” when it contains less than 100 ppm of gluten [4]. Gluten occurs naturally in some ingredients used in food production, and it is also added to food for its technological properties. Additionally, those following this diet should pay particular attention to processed foods that may contain traces of gluten due to cross-contamination. The presence of gluten proteins in naturally gluten-free foods is possible for two reasons. The first is the conscious use of wheat, rye, barley or products based on them. The second reason is cross-contamination, which appears to be the greatest risk. The consumption of gluten-free or naturally gluten-free foods has steadily increased in recent years [8]. Citing Fajrado et al., global market data forecasts GF product sales to grow at a compound annual growth rate of 7.6% from 2020 to 2027 [9]. This is due to numerous diseases in which wheat, barley and/or rye proteins play a key role. There are currently three conditions that require GFD: wheat allergy (WA), non-celiac gluten sensitivity (NCGS) and celiac disease (CD) [2]. Further, of particular importance is the cutaneous manifestation of celiac disease, which is dermatitis herpetiformis, known as Duhring’s disease (DH), which is caused by gluten sensitivity. The incidence of DH varies from 0.4 to 3.5 per 100,000 people per year and affects between 11.2 and 75.3 per 100,000 people in the United States and Europe [10]. Although gluten-related disorders (GRDs) affect about 10% of the general population, gluten-free products are also preferred and perceived as healthier and consumed by people without symptoms related to gluten disorders [1,11,12]. However, according to published research results, gluten-free diets are deficient in many essential nutrients, such as protein, fiber, vitamins and minerals, and they are higher in saturated fat, carbohydrates and salt compared to gluten-containing products. These qualities appear to promote the development of metabolic diseases in CD patients [3,12,13,14]. The final inconvenience of a gluten-free diet is its high cost [13,15,16]. According to Myhrstad et al., the cost of this diet ranges from 46 to 443% more than a regular diet [13], and Fry et al. reported that gluten-free products had a 159% higher price compared to regular gluten-containing foods [15]. Many scientists believe that the choice of naturally gluten-free raw materials is the best alternative both in terms of health and economy [3,17]. The most prominent autoimmune gluten-related disorder is CD, which is increasing worldwide in incidence by 1–2% [18,19,20]. Additionally, NCGS without CD is an immune reaction to gluten, as well as fructans or amylase trypsin inhibitors [21]. Furthermore, wheat is not recommended for people who have been diagnosed with an IgE-dependent WA. However, due to difficulties related to the availability of certain product groups (bread), it is recommended to adhere to the GFD [8]. In recent years, attention has been paid to anaphylaxis caused by the consumption of wheat proteins and the exercise cofactor (WDEIA, exercise-dependent anaphylaxis induced by wheat) [22]. It should be remembered that exercise-induced anaphylaxis may last up to 6 h after gluten consumption [8,23]. This fact underlines the importance of being honest with patients about the possible presence of gluten proteins in food, as their consumption may have significant impacts on certain people’s health. Food producers are obliged to inform consumers about the possible presence of gluten protein fractions in their products [24]. Due to the fact that people with GFD do not always comply with the dietary recommendations resulting from gluten-related diseases [25,26,27,28,29,30], in our own research, food products labelled as “gluten-free” and also those that are naturally gluten-free, without any information on the product label about the possible presence of gluten proteins, were assessed. The considerations above draw attention to the need to assess the degree of gluten contamination of naturally gluten-free food widely available in Poland, which does not contain wheat in its composition on the label.

## 2. Results and Discussion

Of all the certified “gluten-free” and naturally gluten-free products used in the research, only 5.8% were found to contain gluten above 20 ppm. The highest result was recorded for organic pizza and casserole seasoning (646 ppm). Another concerning result has been noted for a processed product intended for self-preparation after pouring boiling water. The average gluten concentration was 200.7 ppm. The ingredients of this product were millet, banana and cocoa, i.e., naturally gluten-free plant materials. As no gluten was found in another product also containing millet and banana, it can be concluded that cross-contamination on the production line might have been possible in the first case.

The first group of products used in our research came from restaurants. These products were not labelled “gluten-free”, but there was no mention of possible gluten protein in the allergen list. This group of products also includes cakes labelled “gluten-free” from patisseries (Table 1). The authors focused on the analysis of products available in catering outlets, which are most often bought and consumed by people with celiac disease. It is noteworthy that the fries and uncontaminated ketchup with gliadin came from two leading chains of bars around the world, which may prove the high standard of services provided. Of the ready-to-eat samples such as fried french fries and ketchups and “gluten-free” ready-made cakes, only one sample was contaminated with gluten at a maximum level of 79.3 ppm, and it was a “cider” type of cake (Table 1). Research has shown that one product (25%) labelled “gluten-free” does not meet the 20 ppm gluten safe requirement. The gluten level in this cake was exceeded by four times. The degree of contamination of products labelled as “gluten-free” varies greatly. Comparing our own results with the results of other authors, it can be concluded that the greatest risk of gluten contamination may be products based on rice flour or starch, because the dough used in others’ research and the bread based on these flours was contaminated 55% above 10 ppm of gliadin (20 ppm of gluten) [5]. It turns out that rice flour, which is one of the most popular flours used by Canadians to increase the amount of fiber in a diet, may contain gluten in the range of 10–48 mg/kg (*n* = 89) [31]. Interestingly, the same research shows that flax seed flour can be contaminated at a level of up to 6134 mg/kg, but flour mix is around (*n* = 54) 321 mg/kg. It turns out that in Italy, Germany, Spain and Norway, the percentage of contaminated certified gluten-free products is the lowest (0 ± 0.5%), whereas in the United States and India, this percentage can be as high as 36% and 32%, respectively [28]. Similar results were obtained for products labelled “gluten-free” purchased in Moscow. Research indicates the presence of gluten protein fractions in 20% of products in the range of 20.3 ± 60.3 mg/kg [32]. In turn, the studies by Farage et al. estimating the presence of gluten contamination in naturally gluten-free meals from food services in Brazil found a total of 2.8% of samples (95% CI: 0.3–5.2%) were contaminated with gluten. In addition, the authors observed that gluten contamination in naturally gluten-free preparations was low in frequency and quantity [33]. Parsons et al. have investigated different practices for gluten cross-contact: gluten-free foods fried in a deep fryer also used for gluten-containing foods, gluten-free bread toasted in a toaster oven also used for gluten-containing bread and popular sandwich spreads applied with a knife used on gluten-containing bread (mayonnaise, jam and peanut butter) [34]. Researchers found that these practices resulted in small amounts of gluten cross-contact, although 93.6% of the results showed no significant cross-contact. Only peanut butter and mayonnaise samples were contaminated with gluten above the limit <20 kg/mg (ppm). It seems, therefore, that as long as the rules of the technological regime are observed, the consumption of food in catering establishments should be safe for people with gluten-dependent enteropathies.

In addition, it should be noted that there are various tests available on the commercial market to check the purity of gluten-free products. Different extraction conditions (80% ethanol, 40% ethanol, SDS/β-mercaptoethanol and 60% ethanol), the complexity of the reference material (gluten, wheat protein, prolamin hydrolysate or gliadin) or type of antibody (R5 mAb, G12, mAb, pAb) mean that the results obtained for different matrices may differ significantly [35]. The method currently recommended and used in control systems in Europe for the analysis of gluten in food is the ELISA R5 Mendez method, which is calibrated against the Gliadin standard of the Prolamin Working Group (PWG) [36]. The monoclonal antibody R5 (mAb) is estimated to be specific for pentapeptides with the amino acid sequences QQPFP and QLPFP [37]. These, in turn, are present in ω1,2-, γ- and α-gliadins as well as some low-molecular-weight glutenin subunits (LMW-GS). The data also show that R5 is responsive to LQPFP, QLPYP, QLPTF, QQSFP, QQTFP, PQPFP, QQPYP, QQQFP and QVQWP. The G12 antibody (AgraQuant Gluten G12 ELISA, Romer Labs, Tulln, Austria) used in own research detects 33-mer peptide from gliadin, which is fraction of “gluten”. The G12 mAb mainly recognizes the QPQLPY and QPQLPF sequences, which are only present in α-gliadins and some ω1,2- and γ-gliadins [37]. The above considerations show that the use of different types of ELISA tests to determine gluten in food may give different results, even several times higher compared to G12 [36,37]. In turn, the research by Hochegger et al. confirm that using the R5 and G12 tests to test the amount of gluten in flour mixes, cookies or cakes or soybean products generally gives similar results. However, for some samples, the results were as much as twice as high with the R5 ELISA [36]. The results obtained in our own research may in some cases be higher than those marked.

The second group of products covered by the study were products containing frozen food (vegetables, ice cream, fries), but mainly food consumed during social gatherings (crisps, alcohol, quick meals). Due to the often difficult access to certified, gluten-free spices, the next group of products were the most commonly used dried spices. The results are shown in Table 2. In this product group, only two positive samples (>above 20 ppm) were recorded. In one of the spices and in a quick dish intended for self-preparation at home, the presence of gluten was at the level of 646.0 and 200.7 ppm, respectively. As much as 95.1% of products in this group were not contaminated with gluten. Recent research results from other authors clearly show that a percentage of naturally gluten-free products not labelled as “gluten-free” do not meet the safety requirements of the restrictive GFD. It should be noted that of the 186 naturally gluten-free products tested in the United States, as many as 19.4% (36/186) did not meet the requirements (>20 ppm) [38]. In India, five samples out of 51 (10%) showed a gluten contamination above 20 ppm [39]. Comparable results (10.1%) were obtained for GF products by Methab et al. [40].

Patients who, because of health issues, are forced to follow a strict gluten-free diet are very often dissatisfied with the quality of gluten-free food, such as bread, pasta or beer [28], which may be the reason they reach out for products based on, for example, malted barley, which is used in the production of beer. Social limitations and low social tolerance are often reasons for not following a GFD, which translates into dealing with long-term complications. Our research shows that malt-based beers are not always a source of gluten proteins. Two of the five beers were gluten-free. In the three positive samples, the mean gluten concentration was 60.4 ppm (Table 3). Technological treatments used in the production of beer (enzymatic treatments, stabilization, raw material selection) could have contributed to the obtained results [6]. It turns out that the use of PVPP (poly(vinylpolypyrrolidone)) or silica gel for stabilization reduces the concentration of gluten in beer to 0.11%, and the use of tannins may deprive beer of immunotoxic epitopes, so that the final product can be called gluten-free [41]. Another method that can be very helpful in the production of wort and beer with reduced or no gluten content is the use of barley grains with a reduced content of immunotoxic proteins for brewing beer. One example of such barley is Pils, which has a toxic protein content of 19,000 ppm, and, for comparison, Carafa barley has 45,000 ppm [42]. Therefore, the proper selection of grain can significantly affect the safety of beer for people with GFD. Perhaps a different type of malt was used in the beers used in our research. Unfortunately, beer producers do not specify such information on the label of their product, so it is difficult to ascertain whether this hypothesis is correct. Informing the consumer of which barley malt the beer was made would be a strong guideline to help them comply more easily with a gluten-free diet. In addition, the expansion of the gluten-free range of products can be carried out through appropriate technological interventions. The use of low-gliadin components, which have been shown to be the most important etiology in gluten-related disorders (α-gliadins and some ω1,2- and γ-gliadins), in the production of gluten-free foods is one option.

Regular dietary consultations are extremely important from the point of view of treatment with a GFD. Repeated advice on how to follow GFD recommendations can increase GFD compliance from 53.3% to 92.4% within 6 months [43]. It turns out that the awareness of people on a gluten-free diet about food that is safe for them is still insufficient. As many as 85% of respondents have a problem with determining whether a given product is safe for people with CD [44], so it can be assumed that some of the forbidden products are consumed consciously or unconsciously by patients. A systematic, summary review the literature from 1980–2007 indicates that strict adherence to the GFD ranged from 42 to 91% [28]. Food cross-contamination, inadequate labelling and social and economic restrictions are factors that make it very difficult to adhere strictly to a GFD. Among 57 people with CD (*n* = 23) and NCDS (*n* = 34), as many as 83% of patients with CD and approximately 68% of patients with NCGS follow a strict GFD (*p* = 0.21) [25]. However, in the same research, some patients diagnosed with CD or NGCS consumed more than 500 mg/kg of gluten daily. The data indicate that the high price of gluten-free-labelled products is determined by the ELISA method used for inspection. Therefore, cheaper and more effective methods of gluten detection are being researched. An interesting alternative to the traditional enzyme-linked immunosorbent assay method seems to be a novel probe that allows fast gluten detection through a simple signaling process with potential use for food control [45]. The sensor developed by the Pla et al. team is made of nanoporous anodic alumina films filled with fluorescent dye and terminated with an aptamer that recognizes gliadin (a soluble gluten protein). In the presence of gliadin, the aptamer sequences dislodge from the surface of the anodic alumina, causing the pores to open and the dye to be delivered [20]. The device has a limit of detection (LOD) of 100 mg kg^−1^ of gliadin, a detection time of about 1 h and good selectivity [20]. 

The COVID-19 pandemic and related home isolation has played a significant role in changing the diets and eating habits of many people. Research conducted during the pandemic showed that among respondents who reported using a GFD due to CD (60.4%), NCGS (29.3%), WA (3.2%) and by their own choice without justification (7.3%), as many as 53.8% of people with GFD consumed food contaminated with gluten [29]. The results of studies published in 2018 showed that only 7% of patients from medical facilities in San Salvador, El Salvador and North America were compliant with the GFD recommendations, which is very worrying [26]. The authors themselves emphasized that their result was the highest ever. In 2017, a cross-sectional survey study conducted in Santa Fe in Argentina found that the percentage of GFD use was 6.3% [46]. In Poland, on the other hand, the situation is less worrying. In research by Czaja-Bulsa and Bulsa, adherence to GFD was investigated with the use of serological tests (tTG). The results of the research showed that more than one-third of 102 patients did not comply with a GFD, but the authors emphasized that this percentage of people is lower and lower every year (40% vs. 26%; *p* < 0.05). The number of children aged 13–18 who did not follow a GFD over 10 years decreased by 14% (54% vs. 40% now; *p* < 0.05) [27]. Therefore, testing uncertified foods as well as certified “gluten-free” foods is particularly important in order to determine the amount of gluten protein consumed by people on a strict GFD and to determine the risk factors associated with gluten protein. However, a study by Weisbrod et al. gives hope to patients on a GFD that foods may not always be as risky for celiac patients as dietary guides suggest [47]. The researchers noted that GF control samples of pasta, bread and muffins were below the detection limit. Gluten was detected in all pasta samples cooked in water used for gluten-containing pasta (33.9 to 115.7 ppm), but rinsing the pasta under running tap water reduced the gluten content to less than 20 ppm. The two samples with detectable gluten had only 5.1 ppm and 17.5 ppm of gluten. Moreover, rinsing pots with water alone after cooking gluten-containing pasta was as effective as scrubbing with soapy water in preventing detectable gluten transfer. Toasting in a common toaster oven was not associated with gluten transfer above 20 ppm; the four samples with detectable gluten had levels ranging from only 5.1 ppm to 8.3 ppm of gluten. Of the 30 muffin samples, 28 had detectable gluten transfer, but only two of them tested >20 ppm [47]. In summary, the use of appropriate technological regimes in meal preparation makes it possible to prepare safe dishes with a gluten content of less than 20 ppm.

Many studies emphasize the importance of healthy and tasty GF products, which the food industry has been invited to produce [17,48]. There are many potential health benefits of consuming gluten-free cereal products and beverages made from cereals or pseudo-cereals. Cereals and pseudo-cereals such as millet, sorghum, teff, quinoa and buckwheat have the potential to increase the nutritional components and health benefits of products such as pasta, bread, cookies, crackers and other unmentioned products that utilize gluten-free grains as their raw ingredients [3,17].

Individuals on a strict gluten-free diet need to pay special careful attention to their nutrition. The GFD diet must not only eliminate toxic gluten fractions, but it also should supply macro- and micronutrients. Patients must remember to including olive oil, legumes, fruits and vegetables in their diet (Mediterranean diet) [3]. An important ingredient of this diet are pseudo-cereals, which are rich in complex carbohydrates, protein, fiber, fatty acids, vitamins and minerals. A very important guideline for individuals following a strict GFD is to avoid processed foods. The most important indication from our research is the fact that the more processed a product is, the higher the chance of gluten protein contamination in the final product (cider—Table 1, quick meals—Table 2).

## 3. Materials and Methods

The tested material was a collection of 57 samples. Among them were 48 naturally gluten-free products that did not contain the information “gluten-free” on the label or information about the possible presence of gluten proteins. The next four samples were cakes labelled as “gluten-free” that were purchased from a cafe. Another five samples were supermarket-bought beers with barley malt. Other samples came from gastronomic points and a supermarket. Eleven samples from gastronomic points were taken for the study, including fries (*n* = 4), ketchup (*n* = 3) added to fries as well as cakes (*n* = 4). In addition, frozen products (*n* = 4), spices (*n* = 12), crisps (*n* = 6), millet-based quick meals (*n* = 3), bars (*n* = 1), spreads (*n* = 3), tea and instant coffee (*n* = 7) and alcohols such as rum, brandy and coffee liqueur (*n* = 4) were tested. All tested products were used for analysis within 24 h of purchase.

The enzyme immunoassay AgraQuant Gluten G12 ELISA test kit (Romer Labs, Tulln, Austria) was used for the analysis. The prepared samples were thoroughly homogenized (OMNITip, Kennesaw, GA, USA). Then, 0.125 g of material was added to 1.25 mL of extraction buffer. The next step was mixing and incubation for 40 min at 50 °C. After cooling to room temperature (20–25 °C), 3.75 mL of 80% ethanol was added, and the mixture was shaken for 60 min. The last stage was centrifugation at 2000 revolutions for 10 min and the removal of the obtained supernatant for analysis. The prepared supernatant was diluted with dilution buffer (1:10). For analysis, 100 µL of ready-made standard and prepared samples were pipetted into the wells of a 96-well plate. Then, everything was incubated for 20 min. After incubation, the plate was washed five times with wash buffer. Then, 100 µL of enzyme-conjugate solution was added and incubated for another 20 min. After this time, the plate was washed, and 100 µL substrate solution was added. The next step was incubation for 20 min, and then adding 100 µL stop solution to the wells. After 10 min, the absorbance at 450 nm was read using a MultiSkan Go reader (Thermo Scientific, Ratastie, Finland). Each sample was analyzed in duplicate. Deionized water was used as a control sample.

According to the AgraQuant^®^ Gluten G12 ELISA test kit guidelines, the LOD = 2 ppm (2 mg kg^−1^) and LOQ = 4 ppm (4 mg kg^−1^). Five standard solutions of gluten (0.0 to 200.0 ppm) were used for the calibration curve (R^2^ = 0.985). The equation of the trendline was:

$$y=1.64+\frac{0.36-1.64}{1+{\left({\left(\frac{x}{210.96}\right)}^{1.21}\right)}^{10}}$$

The Statistica 13.1 software package by StatSoft^®^ (Krakow, Poland) was used for statistical analysis. The minimum and maximum value, median, upper and lower quartile, arithmetic mean and standard deviation were calculated.

## 4. Conclusions

Past research has shown that most randomly selected foods are gluten-free and could easily be labelled “gluten-free”, but this is not the case. Our research clearly shows that many manufacturers, although they do not monitor their products for the presence of gluten in them, offer safe products. Nevertheless, it should be emphasized that due to a lack of information on product labels as to whether they are “gluten-free”, they cannot be recommended for people with celiac disease, as cross-contamination in the production plant may occur depending on the batch of the product. Therefore, there is a strong need to expand the frequency of testing by food manufacturers for the presence of gluten in their products, so that the number of products approved for people on a gluten-free diet continues to increase. Moreover, it is necessary to increase the frequency of routine monitoring of food for the presence of gluten by producers and to provide reliable information to consumers about gluten content in order to increase the availability of certified products that are safe for people on a gluten-free diet.

## Figures and Tables

**Table 1:** The level of gluten contamination in products purchased in gastronomic point (ppm).

Product	Positive Samples	Min	Max	Mean	STD	Me	Q_25_–Q_75_
Fries	0/4 (0%)	nd	nd	nd	nd	nd	nd
Ketchup	0/3 (0%)	nd	nd	nd	nd	nd	nd
Cake *	1/4 (25%)	nd	79.26	19.82	39.63	nd	nd

STD—standard deviation, Me—median, Q_25_–Q_75_—interquantile range, *—labelled as “gluten free”, nd—not detected.

**Table 2:** The level of gluten contamination of naturally gluten-free products that do not have an indication on the label about the possible presence of gluten proteins (ppm).

Product	Positive Samples	Min	Max	Mean	STD	Me	Q_25_–Q_75_
Fries	0/1 (0%)	nd	nd	nd	nd	nd	nd
Frozen vegetables	0/2 (0%)	nd	nd	nd	nd	nd	nd
Cream ice	0/1 (0%)	nd	nd	nd	nd	nd	nd
Spices	1/12 (8.3%)	nd	646.00	53.83	186.48	nd	nd
Crisps	0/6 (0%)	nd	nd	nd	nd	nd	nd
Express dishes based on millet and vegetables	1/3 (33.3%)	nd	200.70	66.90	115.87	nd	nd
Strawberry products (jam, bars)	0/3 (0%)	nd	nd	nd	nd	nd	nd
Vegetable paste	0/1 (0%)	nd	nd	nd	nd	nd	nd
Artificial honey	0/1 (0%)	nd	nd	nd	nd	nd	nd
Drinks (tea, instant coffee)	0/7 (0%)	nd	nd	nd	nd	nd	nd
Alcohols	0/4 (0%)	nd	nd	nd	nd	nd	nd

STD—standard deviation, Me—median, Q_25_–Q_75_—interquantile range, nd—not detected.

**Table 3:** The level of gluten contamination of beers containing barley malt (ppm).

Product	Positive Samples	Min	Max	Mean	STD	Me	Q_25_–Q_75_
Beer	3/5 (60%)	nd	90.23	60.38	40.83	75.65	37.59–83.17

STD—standard deviation, Me—median, Q_25_–Q_75_—interquartile range, nd—not detected.

## Data Availability

Not applicable.

## Sample Availability

Samples are not available from the authors.
